# Laugh and Grow Fat

**Published:** 1874-12

**Authors:** 


LAUGH AND GROW FAT.—"Laugh and Grow Fat" is quite a venerable adage, and Sterne tells us that every time a man laughs, he adds something to his life. An eccentric philosopher of the last century used to say that he not only liked to laugh himself, but to see and hear laughter. Laughter is good for the health, a provocative to appetite, and a friend to digestion. Dr. Sydenham said the arrival of a merry-andrew in a town is more beneficial to the inhabitants than twenty asses loaded with medicines. Though, as Shakespeare avers, all m[illegible] and women are players, yet we little know the state of mind in which they play their parts.

